# Supplementary material for: The impact of visually simulated self-motion on predicting object motion–A registered report protocol
Source: PLoS One. 2023 Jan 30;18(1):e0267983. doi: 10.1371/journal.pone.0267983 (PMC9886253; doi:10.1371/journal.pone.0267983)
Supplement: S1 Appendix — (DOCX) [file pone.0267983.s001.docx]

## Appendix A

Since we assume, for the sake of visualizing our predictions and performing the power analysis, that self-motion impacts the perceived speed equally for both tasks, we first generate parameters indicating biases and variability differences for each participant: for each participant separately, we draw the effect of self-motion in the opposite direction of the target on accuracy from a normal distribution with a mean of 20% of the presented visual self-motion and a standard deviation of 30%. This reflects the rather noisy effect we found in our previous study [1]. For the impact of self-motion in the opposite direction of the target, we draw the values from a normal distribution with a mean of 20% and a standard deviation of 30%. While we did not find such effect previously, the higher self-motion speeds in this study help raise the power and should allow us to find effects for which our previous study did not have enough power. In line with the previous study, no effects were assumed for self-motion in the same direction as the object.

**Modelling the speed estimation task** – To model participant performance, we closely follow the procedure outlined by Jörges [2]: we first generate means and standard deviations for (cumulative Gaussian) psychometric functions for each condition and participant. We compute the means by taking the presented speed of the stimulus (4, 5, or 6 m/s) and adding the effect of self-motion on perceived speed that we have drawn before for each participant. For the standard deviations, we part from a Weber Fraction of 10% for speed judgements commonly reported in the literature [39], and draw the Weber Fractions for each participant from a normal distribution with a mean of 10% and a standard deviation of 1.5%, which corresponds to between-participant variability in their general ability to discern speeds, and convert these values into standard deviations. We then add the value chosen above for the effect of self-motion as a fraction of the presented speed. As a next step, we choose 70 speed values for each condition which our staircase procedure is likely to present for each participant. We achieve this by drawing values from Cauchy distributions with their location parameters at the presented speed and their scale parameter at 10% of the presented speed. We then compute the probability of each participant for each condition and each simulated presented stimulus speed to judge that the ball cloud is faster than the single ball, and use these probabilities to draw binary yes/no answers from the Bernoulli function. Finally, we apply the process outlined under Data analysis plan to obtain simulated PSEs and JNDs for each participant and condition.

**Modelling the prediction task** – For the prediction task, our model for time-to-contact is based on the physical equation for distance from time and speed; time-to-contact can then be estimated from the ball’s speed and the distance between its point of disappearance and the target rectangle.

| $t_{extrapolated}=d_{perceived}/v_{perceived}$ | (3) |
| --- | --- |

We use the same simulated participants as for the speed estimation task (i.e., with the same PSEs and JNDs, and with the same parameters for the effect of self-motion on PSEs and JNDs). We further assume here that the distance between observer and stimulus is either estimated correctly, or it is misestimated consistently, such that the effect on perceived speed and perceived distance cancel out, and that this distance is estimated with a Weber Fraction of 5% [40]. We then draw a (simulated) perceived distance for each trial and participant from a normal distribution with its mean at the correct distance, and a standard deviation that corresponds to this Weber Fraction of 5%. For the perceived speed, we add each participants self-motion effect on their personal PSE and JND, respectively, and then draw a (simulated) perceived speed from a normal distribution that uses the PSE for each participant and trial as mean and the JND for each participant and trial as standard deviation. Finally, we obtain the simulated extrapolated duration by dividing the simulated distance by the simulated speed for each participant and trial and subtract the occlusion duration to obtain the timing error.

**Reference**

1. Jörges B, Harris LR. Object speed perception during lateral visual self-motion. Attention, Perception, Psychophys. 2021;84: 25–46. doi:10.3758/s13414-021-02372-4

2. Jörges B. Data Analysis and Power Simulations with General Linear Mixed Modelling for Psychophysical Data – A Practical , R-Based Guide. PsyArXiv. 2021. doi:10.31234/osf.io/ack8u
